# Large sensitivity in land carbon storage due to geographical and temporal variation in the thermal response of photosynthetic capacity

**DOI:** 10.1111/nph.15100

**Published:** 2018-04-10

**Authors:** Lina M. Mercado, Belinda E. Medlyn, Chris Huntingford, Rebecca J. Oliver, Douglas B. Clark, Stephen Sitch, Przemyslaw Zelazowski, Jens Kattge, Anna B. Harper, Peter M. Cox

**Affiliations:** ^1^ College of Life and Environmental Sciences University of Exeter Exeter EX4 4RJ UK; ^2^ Centre for Ecology and Hydrology Wallingford OX10 8BB UK; ^3^ Department of Biological Sciences Macquarie University North Ryde NSW 2109 Australia; ^4^ Hawkesbury Institute for the Environment Western Sydney University Locked Bag 1797 Penrith NSW 2751 Australia; ^5^ Centre of New Technologies University of Warsaw Banacha 2c 02‐097 Warsaw Poland; ^6^ Environmental Change Institute University of Oxford South Parks Road Oxford OX1 3QY UK; ^7^ Max Planck Institute for Biogeochemistry Hans‐Knöll‐Str. 10 D‐07745 Jena Germany; ^8^ German Centre for Integrative Biodiversity Research (iDiv) Halle‐Jena‐Leipzig Deutscher Platz 5e 04103 Leipzig Germany; ^9^ College of Engineering, Mathematics and Physical Sciences University of Exeter Exeter EX4 4QF UK

**Keywords:** geographical variation of the temperature response of *V*_cmax_ and *J*_max_, modelling photosynthesis, temperature response of photosynthetic capacity, thermal acclimation, tropics, *V*_cmax_

## Abstract

- Plant temperature responses vary geographically, reflecting thermally contrasting habitats and long‐term species adaptations to their climate of origin. Plants also can acclimate to fast temporal changes in temperature regime to mitigate stress. Although plant photosynthetic responses are known to acclimate to temperature, many global models used to predict future vegetation and climate–carbon interactions do not include this process.
- We quantify the global and regional impacts of biogeographical variability and thermal acclimation of temperature response of photosynthetic capacity on the terrestrial carbon (C) cycle between 1860 and 2100 within a coupled climate–carbon cycle model, that emulates 22 global climate models.
- Results indicate that inclusion of biogeographical variation in photosynthetic temperature response is most important for present‐day and future C uptake, with increasing importance of thermal acclimation under future warming. Accounting for both effects narrows the range of predictions of the simulated global land C storage in 2100 across climate projections (29% and 43% globally and in the tropics, respectively).
- Contrary to earlier studies, our results suggest that thermal acclimation of photosynthetic capacity makes tropical and temperate C less vulnerable to warming, but reduces the warming‐induced C uptake in the boreal region under elevated CO_2_.

## Introduction

The response of plant productivity to climate change is a key uncertainty in Earth system models (ESMs; Friedlingstein *et al*., 2006). It has been shown that one of the largest components of this uncertainty is related to the temperature sensitivity of photosynthesis (Matthews *et al*., 2007; Booth *et al*., 2012). Net photosynthetic CO_2_ uptake typically responds to temperature following a peaked relationship, with an optimum temperature between 15 and 35°C (Berry & Bjorkman, 1980). Because the response of photosynthesis to warming depends on whether the prevailing leaf temperature is above or below the optimum, the modelled feedback to climate from land surface carbon (C) uptake is highly sensitive to the value assumed for this optimum temperature (Booth *et al*., 2012).

Accurately representing the temperature response of photosynthesis in ESMs is complex, because the optimum temperature is known to vary both temporally and geographically. Plants growing at low temperatures typically attain their maximum photosynthetic capacity at lower temperatures than do plants growing at warmer temperatures (Berry & Bjorkman, 1980). This variation reflects short‐term acclimation processes as well as longer‐term processes such as genetic adaptation of species to a particular location and/or geographical variation in species composition (Yamori *et al*., 2013; Vanderwel *et al*., 2015). Common‐garden experiments demonstrate that there is a genetic component to the temperature response of photosynthesis, with species or provenances originating from cool environments commonly showing lower optimal temperatures than those originating from warmer environments (Ferrar *et al.,* 1989; Cunningham & Read, 2003; Reich *et al*., 2015; Vårhammar *et al*., 2015). In addition, many plants have a degree of plasticity in temperature sensitivity related to the range of temperatures to which the foliage is exposed. Over short timescales (days, months, up to seasons), plants can adjust their photosynthetic thermal optimum to enable more efficient photosynthesis and potentially maximize C uptake (Berry & Bjorkman, 1980; Ehleringer & Cerling, 1995; Hikosaka *et al*., 2007; Yamori *et al*., 2009; Smith & Dukes, 2017). This fast temporal adjustment of the temperature response driven by a change in growth temperature is known as thermal acclimation (Yamori *et al*., 2013).

Although there is a significant literature documenting variation in the optimal temperature of photosynthesis dating back to the 1970s (e.g. Slayter & Morrow, 1977; Berry & Bjorkman, 1980), most ESMs and dynamic global vegetation models (DGVMs) continue to represent the response of photosynthesis to temperature in a very simple way. Some models use a single temperature response function for all C_3_ and C_4_ species (Wang *et al.,* 2011). Other models prescribe different temperature response curves for tropical, temperate and boreal plant functional types (PFTs), capturing broad geographical variability but ignoring the possibility of thermal acclimation or interspecific differences within biomes (Arora, 2003; Sato *et al*., 2007; Clark *et al*., 2011; Harper *et al*., 2016). Another approach is to vary the optimum temperature based on multi‐annual mean temperature (Krinner *et al*., 2005); this approach captures spatial variability but not thermal acclimation.

Suitable algorithms to represent thermal acclimation of photosynthetic capacity have only emerged relatively recently (Kattge & Knorr, 2007; Friend, 2010; Lin *et al*., 2013; Scafaro *et al*., 2017), principally because most early experimental studies only measured acclimation effects on net photosynthesis (Smith & Dukes, 2013). However, DGVMs require information on acclimation effects for the individual underlying processes that determine the overall temperature response of photosynthetic uptake, including biochemical, respiratory and stomatal regulation (Hikosaka *et al*., 2006; Lin *et al*., 2012). Such measurements are time‐consuming to make and hence are rarer in the literature. Furthermore, models require a synthesis of multiple thermal acclimation datasets that can provide across PFT and biome variations, but such syntheses need to provide mathematical descriptions of each of the individual components of the photosynthesis temperature response in a form that can be used in global models.

The most robust parameterization describing photosynthetic temperature acclimation for C_3_ species as a whole is the study by Kattge & Knorr (2007). Their study focuses on the biochemical component of acclimation. These authors collated and analysed data from multiple studies on the temperature response of the two main biochemical traits underlying the performance of C_3_ photosynthesis in the biochemical model of photosynthesis proposed by Farquhar *et al*. (1980): the maximum carboxylation rate (*V*_cmax_) and potential regeneration rate (*J*_max_) of Ribulose Bisphosphate. Kattge & Knorr (2007) found relationships between the optimum temperatures of *V*_cmax_ and *J*_max_ (*T*_opt,V_ and *T*_opt,J_) and growth temperature, *T*_growth_, defined as the average air temperature during the month before the measurements. Furthermore, they also identified a relationship between the ratio of *J*_max_ to *V*_cmax_ at 25°C (J : V ratio) and *T*_growth_. These three relationships form the core of their acclimation algorithms, from here onwards termed the KK07 algorithms.

The KK07 algorithms are relatively simple to implement into any photosynthesis scheme, including those in DGVMs. They have already been implemented by Ziehn *et al*. (2011), Arneth *et al*. (2012), Chen & Zhuang (2013), Lombardozzi *et al*. (2015) and Smith *et al*. (2016). The latter two studies used the KK07 formulation to quantify the combined impacts of incorporating geographical variability and thermal acclimation of photosynthetic capacity and respiration under a future climate change scenario. Smith *et al*. (2016) reported that accounting for thermal acclimation reduces the simulated carbon sensitivity of terrestrial ecosystems to climate.

However, there are a number of subtleties involved in implementing these algorithms, which may have important implications for model outcomes. First, variation of the J : V ratio with *T*_growth_ can be implemented in several ways with quite different results for simulated photosynthesis. Previous modelling studies have implemented this shift by reducing *J*_max_ at 25°C with warming (Arneth *et al*., 2012; Lombardozzi *et al*., 2015; Smith *et al*., 2016). However, a change in ratio could equally well be achieved by increasing *V*_cmax_ at 25°C with warming (as observed by Lin *et al*., 2013), or by changing both *J*_max_ and *V*_cmax_ at 25°C. The simulated effects of warming on photosynthesis can be very different under each of the above scenarios (Lin *et al*., 2012): reducing *J*_max_ alone is likely to reduce total photosynthesis, whereas increasing *V*_cmax_ alone is likely to increase total photosynthesis overall. KK07 did not find a statistically significant growth temperature effect on either *J*_max_ or *V*_cmax_ at 25°C, suggesting that the approach, with both parameters changing to a small extent, may be the most likely. In the current study, we took this approach, and contrast our results with those of previous studies using alternative implementations.

Second, the KK07 algorithms do not characterize short‐term acclimation alone. The data used in their study cover 36 species, including broad‐leaved trees, coniferous trees, shrubs and herbaceous plants, mostly from temperate regions, under a wide range of growing conditions, including chamber experiments and a wide range of geographical locations. Therefore, the KK07 algorithms incorporate elements of long‐term variation in temperature responses due to geographical gradients in growth temperature as well as thermal acclimation over short‐term (days up to months or seasonal) changes in growth temperature at individual locations. It is not known to what extent these two sets of processes contribute to the overall observed response: at this point in time, no data syntheses have attempted to robustly determine the relative importance of short‐term acclimation and long‐term geographical variation of the thermal responses of photosynthetic capacity, nor whether the mechanisms behind these responses to growth temperature (geographical gradients, temporal changes) are the same.

Whether the overall changes in temperature response observed by KK07 are assumed to be due principally to long‐term geographical variation or short‐term thermal acclimation could have significantly different consequences for predicted land C storage under warming scenarios. To explore the relative importance for the two sets of processes, we implemented the KK07 algorithms in two different ways, representing two extremes: (1) assuming that long‐term geographical variation dominates, and there is no short‐term acclimation, the parameters were assumed to vary geographically only, as a function of the local mean temperature during the pre‐industrial period; (2) assuming that short‐term acclimation dominates, the parameters *T*_opt,V_, *T*_opt,J_ and J : V were assumed to vary both geographically and temporally, as a function of the mean temperature of the previous month.

We hypothesized that for the 20^th^ Century and present‐day, accounting for the differences in temperature responses of photosynthetic capacity among plants growing in thermally contrasting habitats (i.e. geographical variation) would have a bigger effect on the land carbon cycle than accounting for short‐term temporal variation (i.e. thermal acclimation). However, we hypothesized that accounting for short‐term thermal acclimation would be more important for predicting future changes in land carbon than during the 20^th^ Century.

In order to test our hypotheses, we implemented the KK07 algorithms in the Joint UK Land Environment Simulator (JULES; Clark *et al*., 2011), the land‐surface scheme used in the UK Hadley Centre ESM, and quantified current and future land C storage. In a further advance on previous studies, we account for climate–carbon cycle feedbacks. JULES is coupled to the computationally efficient climate–carbon cycle model IMOGEN (Huntingford *et al*., 2010) driven with patterns of climate change that emulate 22 full‐complexity global climate models (GCMs). Using this range of climate change projections allows representation of full uncertainty in future physical climate responses to be introduced into our coupled C cycle simulations.

## Materials and Methods

### Variation in the temperature response of photosynthetic capacity

We used the KK07 algorithms which comprise three empirical relationships between growth temperature and the temperature responses of maximum carboxylation rate (*V*_cmax_) and potential regeneration rate (*J*_max_) of Ribulose Bisphosphate (μmol m^2^ s^−1^). According to these relationships, optimum temperatures of *V*_cmax_ and *J*_max_ (*T*_opt,V_ and *T*_opt,J_) increase by 0.44°C and 0.33°C per degree increase in growth temperature, respectively, and the J : V ratio at 25°C decreases by 0.035°C per degree increase in growth temperature (*T*_growth_), defined by KK07 as the average air temperature during the month before the measurements. These relationships were implemented as follows. The temperature responses of *J*_max_ and *V*_cmax_ are represented in Eqn (Eqn 1).

(Eqn 1) $$k_{\;T}=k_{25}\exp\left[H_{a}\frac{\left(T_{l}-T_{\mathrm{ref}}\right)}{T_{\mathrm{ref}}RT_{l}}\right]\frac{1+\exp\left[\frac{T_{\mathrm{ref}}\Delta S-H_{d}}{T_{\mathrm{ref}}R}\right]}{1+\exp\left[\frac{T_{l}\Delta S+H_{d}}{T_{l}R}\right]}$$

*k*_T_ (μmol m^2^ s^−1^) is either *J*_max_ or *V*_cmax_ at leaf temperature *T*_l_ (K); *k*_25_ (μmol m^2^ s^−1^) is the base rate of *J*_max_ or *V*_cmax_ at the reference temperature *T*_ref_ of 25°C (K); *H*_a_ and *H*_d_ (J mol^−1^) are activation and deactivation energies, respectively, that describe the rate of increase and decrease below and above the optimum temperature *T*_opt_, respectively; Δ*S* (J mol^−1^ °C^−1^) is an entropy factor; and *R*, the universal gas constant (8.314 J K^−1^).) For this equation, *T*_opt,V_ and *T*_opt,J_ are given by:

(Eqn 2) $$T_{\mathrm{opt}}=\frac{H_{d}}{\Delta S-R\log_{e}\left[\frac{H_{a}}{H_{d}-H_{a}}\right]}$$

Following KK07, the geographical variation and thermal acclimation of the temperature responses of *J*_max_ and *V*_cmax_ were represented by varying the parameter Δ*S* with *T*_growth_ according to Eqn (Eqn 3):

(Eqn 3) $$\Delta S_{i}=a_{i}+b_{i}\times T_{\mathrm{growth}}$$

Sub index *i* refers to *J*_max_ or *V*_cmax_. Acclimation parameters (*a*_*i*_ and *b*_*i*_) for each of these terms were derived by KK07 and can be found in Supporting Information Table S1. In addition, KK07 showed the J : V ratio to decline with *T*_growth_ following Eqn (Eqn 4) (*a* and *b* are in Table S1)

(Eqn 4) $$JV=a+b\times T_{\mathrm{growth}}$$

In order to implement this relationship, we assumed that the total amount of leaf nitrogen (N) allocated to photosynthesis remains constant. Thus, increasing *J*_max_ requires a decrease in *V*_*c*max_ according to the nitrogen requirements of both processes. Following Medlyn (1996), we estimated the trade‐off between total leaf N allocated (*N*_tot_) to photosynthesis via *J*_max_ and *V*_cmax_ as a constant value of 5.3 : 3.8. Thus, we assumed the J : V ratio varied with *T*_growth_ following Eqn (Eqn 5):

(Eqn 5) $$V_{\mathrm{cmax}}/3.8+J_{\max}/5.3=N_{\mathrm{tot}}=\text{constant}$$

We applied the KK07 algorithms to C_3_ plants only and owing to a lack of similar data we did not consider geographical and thermal acclimation of C_4_ plants.

### Land surface model and climate system

Our study used JULES (Clark *et al*., 2011) to simulate C stocks and fluxes in vegetation and soils over time. The original JULES C_3_ photosynthesis model from Collatz *et al*. (1991) was replaced by the Farquhar *et al*. (1980) C_3_ photosynthesis model in order to use the same equations and parameter values as in KK07. Then, we implemented variable temperature responses as described above. Details of photosynthesis model equations, leaf‐ to canopy‐ to grid‐level scaling, dynamic vegetation, stomatal conductance, leaf and plant respiration in JULES are included in Notes S1.

We used JULES within a computationally efficient climate–carbon cycle system (IMOGEN; Huntingford *et al*., 2010) for the period 1860–2100. This system is based on pattern‐scaling of climate model output and estimates surface meteorology against overall global warming levels (see Notes S2 for further details).

### Simulations

We performed JULES–IMOGEN simulations using three model configurations (Table 1). We first applied the KK07 algorithms to represent only the geographical variation of the temperature response of photosynthetic capacity due to long‐term processes, that is, without inclusion of acclimation to short‐term temporal changes in temperature (i.e. over days, seasons or yearly); these simulations were denoted *Geog*. This configuration assumes that global geographical patterns of photosynthetic capacity are due to inherent differences in temperature responses among plants growing in thermally contrasting habitats, with these plant types being unable to acclimate to sustained changes in *T*_growth_. Therefore, we assumed that temperature responses relate to climate of origin and took pre‐industrial climate as its proxy, taken as 1901–1910 from the Climate Research Unit (CRU) dataset (New *et al*., 2000). Here, geographical variation in *T*_opt,V_ and *T*_opt,J_ and J : V was estimated by applying Eqns (Eqn 1), (Eqn 2), (Eqn 3), (Eqn 4), (Eqn 5) to monthly‐mean pre‐industrial air temperatures (Fig. S1). In *Geog, T*_growth_ were set to the mean local annual air temperatures for gridcells in latitudes between 30°N and 30°S, and to mean monthly air temperature of the three warmest months of the year elsewhere.

**Table 1:** Summary of model configurations

Model configuration^a^	*T*_growth_	Physiological interpretation
*Geog*	Based on a pre‐industrial reference period 1901–1910. In the tropics *T*_growth_ is fixed to mean annual monthly *T*_air_. Elsewhere *T*_growth_ is defined as mean monthly *T*_air_ of the warmest quarter of the year.	The temperature response of photosynthetic capacity varies geographically due to inherent differences in temperature responses among plants growing in thermally contrasting habitats due to extensive long‐term physiological and biochemical plant adjustments to large geographical variations in temperature. In this case, plants are unable to acclimate to sustained changes in growth temperature.
*Geog +Acclim*	*T*_growth_ is mean current month *T*_air_. It varies spatially and temporally.	The temperature response of photosynthetic capacity varies geographically and temporally. It is assumed that there are no inherent differences in temperature responses of photosynthetic capacity across plants but all can acclimate to changes in growth temperature.
*Ctrl*	na	There are no differences in the temperature response of photosynthetic capacity across plants and there is no geographical long‐term variability nor thermal acclimation, therefore all plant types are represented with a single temperature response function and parameters.

na, not applicable.

a All configurations use a common underlying framework, the KK07 algorithms (Eqns (Eqn 1), (Eqn 2), (Eqn 3), (Eqn 4), (Eqn 5)) but applied in a different manner.

Second, we applied the KK07 algorithms to represent the combined geographical long‐term variation and thermal acclimation effects, denoted *Geog+Acclim*. This configuration assumed no inherent differences in temperature responses of photosynthetic capacity across plants but all can acclimate to changes in *T*_growth_. In *Geog+Acclim*,* T*_opt,V_ and *T*_opt,J_ and J : V were both dynamic in time (monthly) and space (gridbox level), varying as a function of the gridbox mean current month air temperatures (*T*_growth_). Finally, we assumed neither geographical long‐term variability nor thermal acclimation in *Ctrl* simulations. Here all C_3_ PFTs were represented with a single temperature response function using values proposed by KK07 with *T*_opt,V_ and *T*_opt,J_ of 32.76°C and 32.12°C, respectively, and a J : V of 1.97.

We performed leaf‐level, ecosystem‐level and global coupled climate–carbon cycle simulations under the three model configurations. Leaf‐level simulations were used for process understanding, ecosystem‐level for evaluation purposes and global simulations for process quantification and projection.

### Leaf‐level simulations

In order to understand projected regional and global predictions, we examined the individual geographical and thermal acclimation effects on the temperature response of light‐saturated gross photosynthetic uptake for sunlit leaves at the leaf level in three randomly selected gridcells for three PFTs based on a land cover map (Poulter *et al*., 2015), within boreal & tundra (shrub), temperate (grassland) and tropical (forest) environments (see Notes S3; Table 2).

**Table 2:** Gridcell coordinates, atmospheric [CO_2_], growth temperature (*T*_growth_) and temperature sensitivity parameters specified on each leaf level simulation (Fig. 1) under pre‐industrial (PI, 1860) and future (2100) conditions for different seasons

Model configuration	Location, PAR (μmol m^−2^ s^−1^)	CO_2_ (ppm)	*T*_growth_ (°C)	*T*_opt_ of *V*_cmax_ (°C)	*T*_opt_ of *J*_max_ (°C)	JV	*V*_cmax_ at 25°C (μmol m^−2^ s^−1^)	*J*_max_ at 25°C (μmol m^−2^ s^−1^)
Tropical broad leaf tree	2.5°N, 60°W	(PI, Future)						
* Ctrl*		286.2, 839.1	na	32.8	32.13	1.97	36.8	72.5
* Geog*	1500	286.2, 809.8	26.3	36.9	34.9	1.7	40.5	67.3
* Geog + Acclim*		286.2, 796.6						
* *Pre‐Industrial			26.3	36.9	34.9	1.7	40.5	67.3
* *Future			31.25	39.5	36.7	1.5	43.0	64.0
Temperate C_3_ grass	40**°**N, 90**°**W	(PI, Future)						
* Ctrl*		286.2, 839.1	na	32.8	32.13	1.97	58.4	115.1
* Geog*	1000	286.2, 809.8	22.54	35.0	33.6	1.8	61.6	110.6
* Geog + Acclim*		286.2, 796.6	*Cold, warm*	*Cold, warm*	*Cold, warm*	*Cold, warm*	*Cold, warm*	*Cold, warm*
* *Pre‐Industrial			11.2, 22.5,	29.4, 35.0	29.7, 33.6	2.2, 1.8	54.7, 61.6	120.2, 110.6
* *Future			13.8, 30.8	30.7, 39.3	30.6, 36.6	2.1, 1.5	56.2, 67.8	118.2, 102.0
Boreal & Tundra Shrub	65**°**N, 105**°**E	(PI, Future)						
* Ctrl*		286.2, 839.1	na	32.8	32.13	1.97	24	47.3
* Geog*	1000	286.2, 809.8	14.1	30.8	30.7	2.1	23.1	48.5
* Geog + Acclim*		286.2, 796.6	*Cold, warm*	*Cold, warm*	*Cold, warm*	*Cold, warm*	*Cold, warm*	*Cold, warm*
* *Pre‐Industrial			2.9, 14.1	25.4, 30.8	26.9, 30.7	2.5, 2.2	20.8, 23.1	51.7, 48.5
* *Future			7.7, 16.7	27.7, 32.1	28.5, 31.6	2.3, 2.0	21.7, 23.8	50.4, 47.6

*Cold* refers to mean values during winter, spring and autumn in the temperate gridcell and to spring and autumn in the boreal & tundra gridcell. *Warm* refers to mean summer time values in temperate and boreal & tundra gridcells.

na, not applicable.

In order to show the implications of the leaf‐level responses at the regional scale, we extracted the gross primary productivity (GPP) for all gridcells for three regions, tropical (30°N < Lat < 30°S), temperate (60°N > Lat > 30°N and 60°S > Lat > 30°S), and boreal and tundra (60°S < Lat > 0°N) from the global JULES–IMOGEN simulations.

### Model evaluation

We performed site‐level simulations with the three model configurations at 22 flux‐sites from Fluxnet (http://fluxnet.fluxdata.org/data/fluxnet2015-dataset/) and four from Brasil‐flux (Restrepo‐Coupe *et al*., 2013; Table S2) representing different ecosystems, using hourly site‐level meteorological forcing. *T*_growth_ in *Geog* and *Geog+Acclim* was estimated as above (Table 1). We used a regression‐based approach to test for the presence of geographical and thermal acclimation effects comparing simulated daily GPP and that derived from flux‐sites. Specifically, we included days when eight or more simultaneous hourly observations and simulations were available with GPP > 1 μmol m^−2^ s^−1^ (i.e. day time values).

Similar to the detection and attribution methods in temperature observations (Huntingford *et al*., 2006; Hegerl *et al*., 2007), regression fits were made by sequentially adding new effects and testing for their presence. Regression coefficient values near unity may indicate that any new modelled effect was both observable in the measurements, and had the correct order of magnitude in its calculation. The regression (Eqn (Eqn 6)) consisted of both a background simulation GPP_BACK_, and additional incremental component to test for ‐ΔGPP_NEW_‐, fitted to GPP observations GPP_OBS_ with two respective regression coefficients *β*_1_ and *β*_2_:

(Eqn 6) $${\text{GPP}}_{\mathrm{OBS}}=\beta_{1}\;{\text{GPP}}_{\mathrm{BACK}}+\beta_{2}\;\Delta {\text{GPP}}_{\mathrm{NEW}}+\varepsilon$$

(*ɛ*, a noise term). Three sets of fits were performed for each site: first, GPP_BACK_ was from *Ctrl*, and ΔGPP_NEW_ was the difference in GPP in *Geog* and *Ctrl* for each timestep, with the ‘Geographical effect’ captured in *β*_2_. In the second fit, GPP_BACK_ also was from *Ctrl*, but instead ΔGPP_NEW_ was the difference in modelled GPP between *Geog+Acclim* and *Ctrl* with the combined ‘Geographical and thermal acclimation effects’ represented by *β*_2_. In the last fit, GPP_BACK_ was from *Geog* and ΔGPP_NEW_ was the difference in GPP between *Geog+Acclim* and *Geog*. Here *β*_2_ represents the ‘Acclimation effect’.

For comparison, simple linear regressions also were performed between GPP_OBS_ and GPP from each of the three model configurations ‘GPP_CONF_’ (*Ctrl*,* Geog*,* Geog+Acclim*) as:

(Eqn 7) $${\text{GPP}}_{\mathrm{OBS}}=\beta \;{\text{GPP}}_{\mathrm{CONF}}+\varepsilon$$

### Global simulations

We performed global JULES–IMOGEN simulations for the 1860–2100 period, forced with climate change patterns and energy balance model parameters that emulate the 22 GCMs used in the IPCC AR4 (Meehl *et al*., 2007). The predicted changes in surface meteorology were added to a baseline period (1901–1910) from the CRU climatology (New *et al*., 2000) as in Huntingford *et al*. (2013). CO_2_ emissions and non‐CO_2_ radiative forcings were taken from the SRES‐A2 business‐as‐usual emissions scenario (Nakicenovic & Swart, 2000). All simulations used an hourly time‐step and a spatial resolution of 2.5° × 3.75°.

The individual ‘Geographical’ and ‘Thermal acclimation’ effects on land C storage were calculated as the difference in change in land C storage between the two relevant sets of simulations over the study period, 1860–2100. This ‘difference in change’ approach explicitly targets the change in C over the study period, taking into consideration differences in initial 1860 conditions across simulations.

(Eqn 8) $$\text{Effect}={\left(C_{{\mathrm{tot}}_{2100}}-C_{{\mathrm{tot}}_{1860}}\right)}_{a}-{\left(C_{{\mathrm{tot}}_{2100}}-C_{{\mathrm{tot}}_{1860}}\right)}_{b}$$

$C_{{\mathrm{tot}}_{2100}}$ and $C_{{\mathrm{tot}}_{1860}}$ (Pg) represent the total amount of C in vegetation and soils at the end of the simulation period (i.e. 2100) and at the start of the simulation period (i.e. 1860), respectively. To estimate the ‘Geographical’ effect, indices ‘*a*’ and ‘*b*’ denote ‘*Geog*’ and ‘*Ctrl*’ simulations, respectively, and to estimate the ‘Acclimation’ effect, ‘*a*’ and ‘*b*’ denote ‘*Geog+Acclim’* and ‘*Geog*’, respectively. Likewise, we estimated the land C enhancement due to individual effects as follows:

(Eqn 9) $$\text{Land C Enhancement}=\frac{{\left(C_{{\mathrm{tot}}_{2100}}-C_{{\mathrm{tot}}_{1860}}\right)}_{a}-{\left(C_{{\mathrm{tot}}_{2100}}-C_{{\mathrm{tot}}_{1860}}\right)}_{b}}{{\left(C_{{\mathrm{tot}}_{2100}}-C_{{\mathrm{tot}}_{1860}}\right)}_{b}}$$

## Results

### Leaf‐level simulations

**Figure 1:**
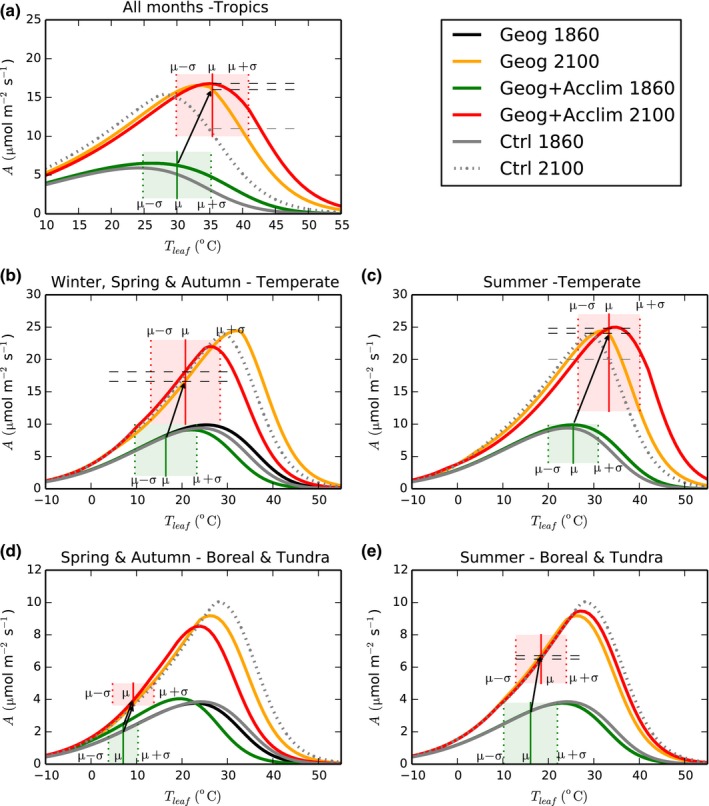
Schematic representation of leaf‐level temperature response of light‐saturated gross photosynthesis by sunlit leaves using *Ctrl*,* Geog* and *Geog+Acclim* configurations on (a) a tropical forest (whole year), (b, c) temperate grassland (spring & autumn and summer months, respectively) and (d, e) shrub in the boreal & tundra region (spring & autumn and summer months, respectively) at pre‐industrial (continuous grey, black and green lines) and year 2100 (dotted grey, yellow and red lines) temperatures using an intermediate model in terms of predicted warming for illustrative purposes (gfdl_cm2). Note the scale differences across plant functional types (PFTs). The mean ± 1 SD (μ ± σ) of daytime hourly leaf temperature are represented in the green and red shaded boxes for the years 1860 and 2100, respectively. The black arrow represents the change in photosynthesis in *Geog* at the mean daytime temperature between 1860 and 2100. The dashed lines represent the extra carbon from acclimation at the mean daytime temperature in 2100.

The leaf‐level response of light‐saturated gross photosynthetic uptake (*A*) to leaf temperature (*T*_Leaf_) is presented in Fig. 1 for the chosen PFTs at different geographical locations under pre‐industrial (1860) and future (2100) atmospheric CO_2_ and *T*_growth_ conditions (Table 2). In all cases *A* at any given *T*_Leaf_ was highest under 2100 conditions due to the CO_2_ fertilization effect on photosynthesis.

**Figure 2:**
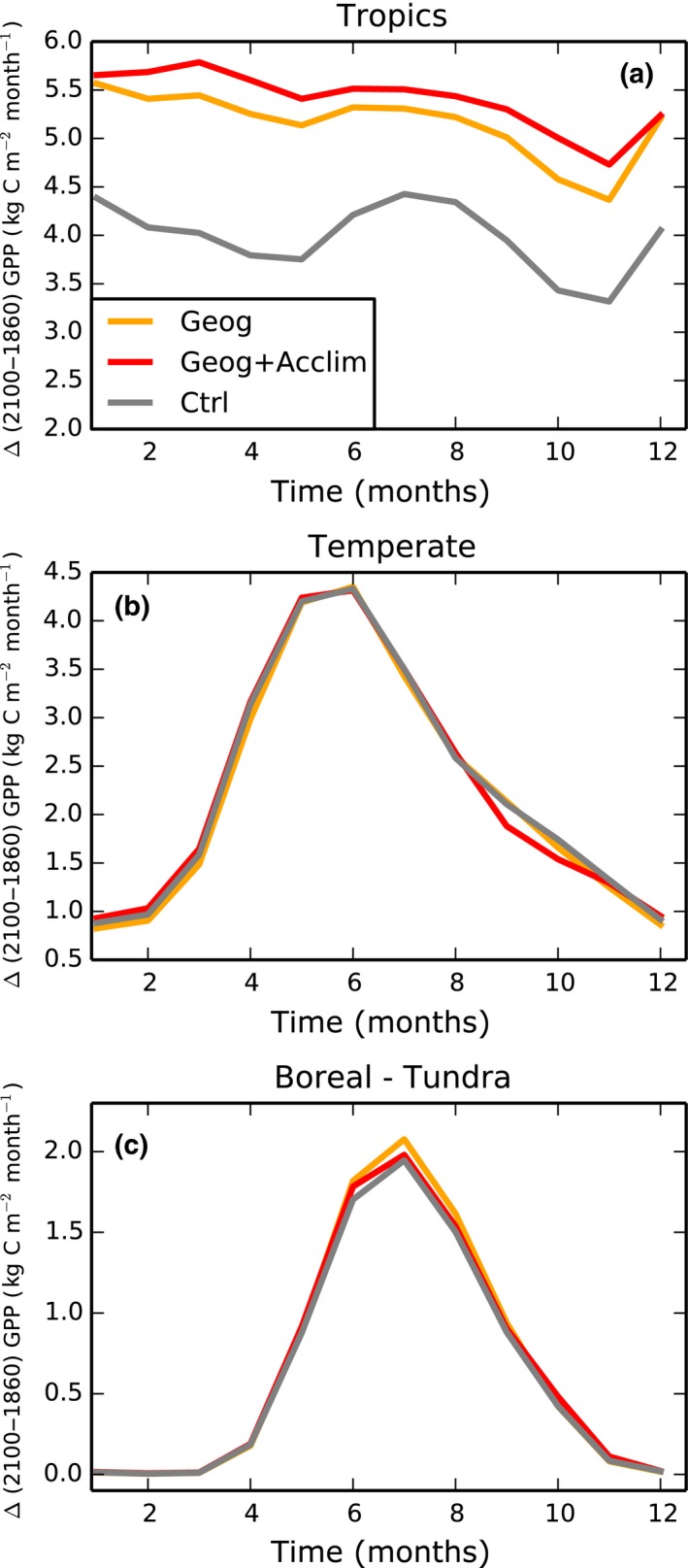
Change in simulated gross primary productivity (GPP) averaged for the (a) tropics, (b) temperate, and (c) boreal and tundra regions over the 1860–2100 period with climate from the gfdl_cm2 model. Note the differences in *y*‐axis scales.

For the tropical broadleaf tree (Fig. 1a), under pre‐industrial conditions, *T*_opt,V_ and *T*_opt,J_ (Table 2) were highest in *Geog*, and in 2100 the highest *T*_opt_ parameters were obtained in *Geog+Acclim*. These values together with variations in *V*_cmax_ and *J*_max_ at 25°C (Table 2) resulted in more photosynthetic uptake under *Geog* than *Ctrl* for the range of simulated day‐time hourly leaf temperatures in 1860 (green shaded box) and in 2100 (red shaded box). Furthermore, in 1860 by definition *Geog* and *Geog+Acclim* are identical having the same *T*_growth_ (Table 1). However, in 2100 there was a benefit from *Geog+Acclim* (red line) over *Geog* (orange line) above a *T*_Leaf_ threshold, located near the optimum temperature for photosynthesis in *Geog+Acclim* (coincides with *T*_Leaf_ above the mean day‐time temperatures, i.e. vertical red line), below which there is no benefit from thermal acclimation. Results for the whole tropical region (Fig. 2a) are similar to those obtained at leaf‐level: highest carbon uptake in *Geog+Acclim* and lowest in *Ctrl*.

Similar results at the leaf‐level were obtained for the temperate C_3_ grassland during summertime conditions (Fig. 1c). However, during the rest of the year (Fig. 1b; Table 2) *T*_opt,V_ and *T*_opt,J_ were lowest in *Geog+Acclim* – due to seasonal acclimation – and highest in *Geog*. In 1860, the three model configurations show similar photosynthetic uptake (Fig. 1b). In 2100 during usual daytime conditions (red shade), *Geog+Acclim* benefitted from having the lowest *T*_opt_ followed by the *Ctrl* simulation and *Geog* obtained the least C uptake. Results for the whole temperate region (Fig. 2b) showed relatively small differences among simulations.

For the boreal and tundra leaf‐level temperature responses, *T*_opt,V_ and *T*_opt,J_ were lowest in *Geog+Acclim* and highest in *Ctrl* (Table 2). Results during spring and autumn (Fig. 1d) were similar to those obtained for the temperate grassland (Fig. 1b). However, there was greater benefit from thermal acclimation in the temperate grassland because it had higher growth and day‐time temperatures than in the boreal & tundra shrub (i.e. compare the location of green and red boxes in Fig. 1b and d). Additionally in 1860 there was a benefit from thermal acclimation for the boreal & tundra shrub. Under future summer‐time conditions (Fig. 1e), *T*_opt_ shifts towards the higher summer air temperatures in *Geog+Acclim*, with parallel changes in photosynthetic capacity at 25°C (increased *V*_cmax_ and decreased *J*_max_; Fig. S2; Table 2). Together these changes result in an inferior plant performance in terms of C uptake at the prevailing leaf temperatures during this season (red line below yellow line in the red shaded box in Fig. 1e). In this case vegetation makes less progress towards its optimum, and subsequently has a reduced enhancement of photosynthesis during summer in the boreal and tundra region (Fig. 2c).

In summary, results demonstrate increased GPP in *Geog* in the tropics and boreal and tundra regions and little effect in the temperate region. This is an expected result as our *Ctrl* simulation uses mean parameters values from the KK07 dataset, composed of mostly temperate data, increased GPP via thermal acclimation under all conditions and regions analysed in 2100 except in the boreal and tundra regions during the summer months.

### Model evaluation for present‐day conditions

**Figure 3:**
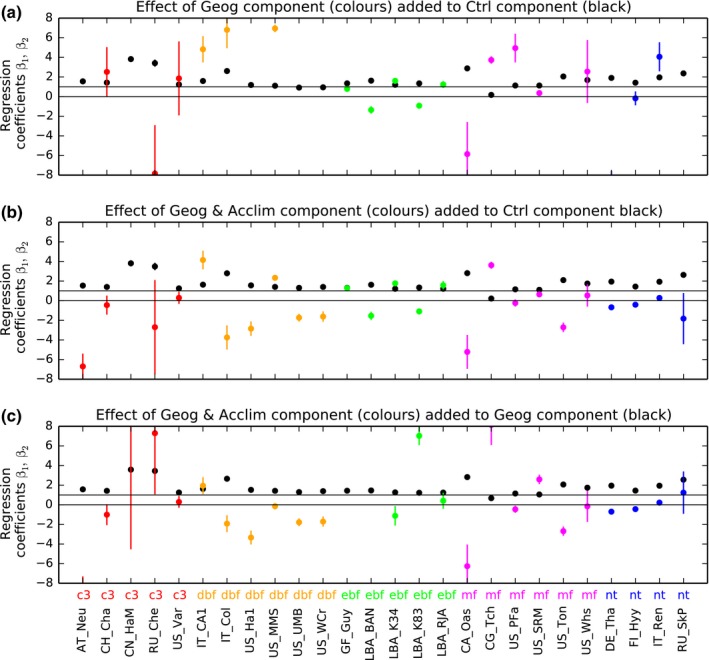
Ecosystem‐level evaluation. Black dots are regression coefficient *β*_1_ (Eqn (Eqn 6)) which in (a) and (b) are the component of *Ctr* simulations to observations, and in (c) are *Geog* simulations. Coloured dots (*β*_2_ in Eqn (Eqn 6)) correspond to additional components, of (a) Geographical effects, (b) Geographical and acclimation effects, and (c) thermal acclimation effects only. Vertical lines correspond to the 95% confidence intervals on regression coefficients. Values of zero and unity are marked; values near unity suggest modelled effects may be observable in the measurements, and that its calculation is of the correct order of magnitude. Some sites had ‘*β*_2_’ values out of the focal range on the vertical axis.

The regression coefficient values of Eqn (Eqn 6), *β*_1_ (black) and *β*_2_ (coloured) dots in Fig. 3 were analysed for their nearness to unity indicating whether the modelled effect was both observable in the measurements, and that its calculation was of the correct order of magnitude. Our most consistent findings are at evergreen forest sites (ebf; Fig. 3a,b) with three *β*_2_ values near to unity, suggesting the geographical effect to be important. No thermal acclimation effect was found decisively at any site.

In comparison the simple linear regressions (Eqn (Eqn 7)) are presented in Fig. S3. For most vegetation types, the *β* values become nearer to unity as geographical effects are included, implying model improvement. However, the annotated RMSE between model and observations do not always show parallel improvements.

### Regional scale

**Figure 4:**
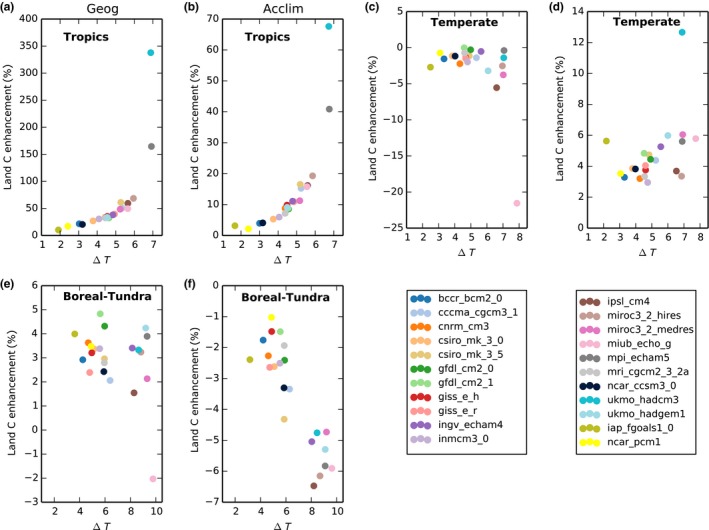
Simulated enhancement of land carbon storage due to (a, c, e) individual geographical and (b, d, f) acclimation effects for 22 global climate models (GCMs) as a function of the change in regional land surface temperature over the study period (Δ*T*). Rows represent regions: tropical (30°N < Lat < 30°S), temperate (60°N > Lat > 30°N and 60°S > Lat > 30°S) and boreal and tundra (60°S < Lat > 60°N) regions. Note the differences in *y*‐axis scales.

**Figure 5:**
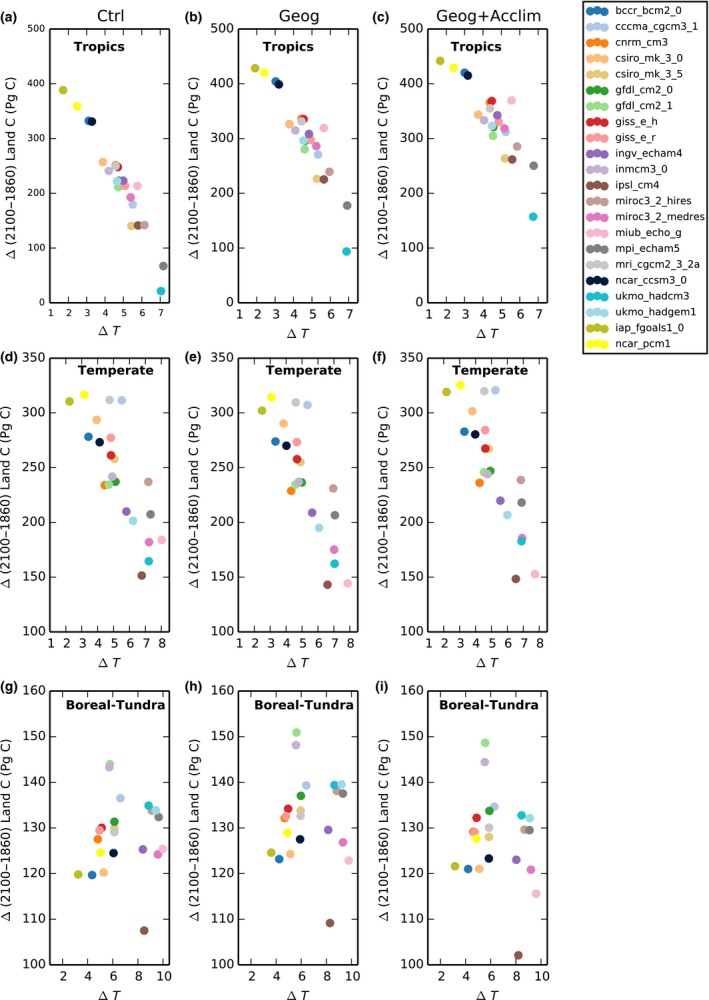
Simulated change in global land carbon over the study period in (a, d, g) *Ctrl*, (b, e, h) *Geog* and (c, f, i) *Geog+Acclim* for 22 global climate models (GCMs) as a function of the change in regional land surface temperature over the study period (Δ*T*). Rows represent regions: tropical (30°N < Lat < 30°S), temperate (60°N > Lat > 30°N and 60°S > Lat > 30°S) and boreal and tundra (60°S < Lat > 60°N). Note the differences in *y*‐axis scales.

Results across climate models show a mean enhancement of land C accumulation in the tropics of 37 ± 15% (range between 10% and 69% when excluding two outliers at 165% and 338%; see Fig. 4a) and 9.6 ± 5.8% (range between 2% and 19% when excluding two outliers at 41% and 67%; see Fig. 4b) between 1860 and 2100 due to geographical and thermal acclimation effects, respectively. In the tropics, the largest enhancement (Fig. 4a,b) corresponded to climate models that predicted the greatest warming and the lowest land C gain over the simulation period (Fig. 5a–c). In the temperate regions, there was a negative geographical effect with a model mean of −2.5% (range between −21.5 and 0%) and a small positive thermal acclimation effect of 4.8% (range between 3 and 13%; Fig. 4c,d). In the boreal and tundra regions, the simulated mean C enhancement due to geographical effects across models was only 3% (range between −2.0 and 4.8%) with one model simulating a loss and there was a reduction in C gain due to thermal acclimation with a mean across models of −3.5% (range between −6.5 and −1%; Fig. 4e,f). This counterintuitive finding was explained by the leaf‐level analysis presented in Figs 1(e)–2(c); these regions did not benefit from acclimation under future summertime conditions.

### Global scale

**Figure 6:**
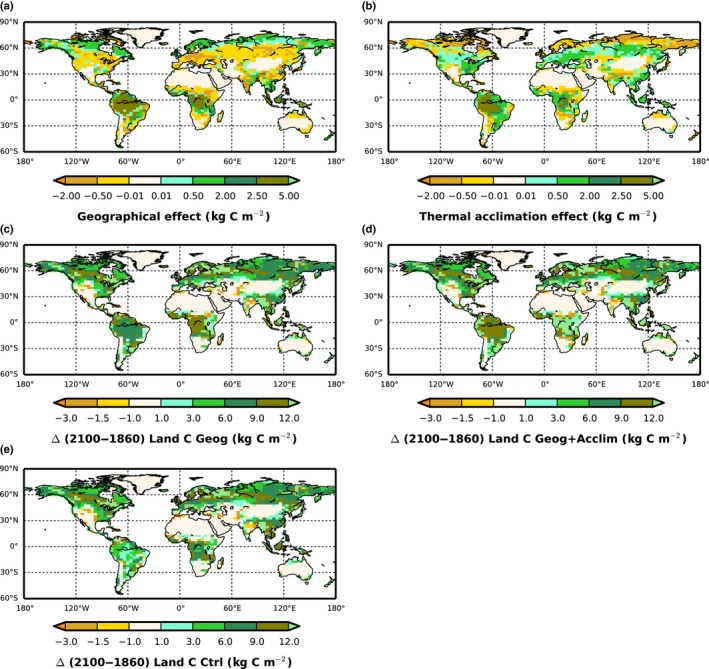
Impact of incorporating (a) geographical variability and (b) thermal acclimatization of temperature sensitivity of photosynthetic capacity on land carbon (C). The (a) Geographical effect was estimated (Eqn (Eqn 8)) as the multi‐model mean change in land C storage (kg C m^−2^) in (c) the *Geog* simulation minus change in the *Ctrl* simulation over the study period (1860–2100). Correspondingly the (a) acclimation effect was estimated as the difference between (d) *Geog+Acclim* and (c) *Geog* simulations. Positive (negative) values represent an increase (decrease) in land C storage.

These results showed a positive geographical effect on C storage in most tropical areas, boreal and tundra regions but negative in the temperate regions and some tropical areas (Fig. 6a). Thermal acclimation enhanced the land C sink in tropical and temperate regions but reduced the sink in some boreal and tundra regions (Fig. 6b). The individual global geographical and acclimation effects, accumulated over different time periods are presented in Table 3. Overall, geographical effects led to a global net enhancement of the land C sink of 78.4 ± 14.8 PgC, approximately double that associated with thermal acclimation (37.9 ± 14.6 PgC) across climate models over the study period (Table 3). Simulated global fields of GPP and land C in 1860 are provided in Table S2.

**Table 3:** Global Land C (in soils and vegetation) accumulated over the specified simulation period on each column, estimated as the difference in total Land C at end and the start of the simulation period

	1860–1899 μ ± σ (range)	1900–1999 μ ± σ (range)	2000–2099 μ ± σ (range)	1860–2100 μ ± σ (range)
Global land C accumulation (Pg)				
* Control*	11.6 ± 0.5 (11–13)	129.6 ± 7 (111–142)	436 ± 113 (183–644)	577 ± 118 (308–797)
* Geog*	11.9 ± 0.5 (11–13)	135.3 ± 6 (118–146)	508 ± 105 (251–684)	656 ± 111 (382–840)
* Geog + Acclim*	11.3 ± 0.5 (11–13)	139.1 ± 6 (124–149)	543 ± 95.0 (323–701)	694 ± 100 (459–860)
Individual effects (Pg)				
* Geographical effect*	0.3 ± 0.07 (0.2–0.5)	6 ± 0.7 (4–7)	72 ± 14 (32–108)	79 ± 15 (37–116)
* Thermal acclimation effect*	−0.6 ± 0.06 (−0.7, −0.5)	4 ± 0.6 (3–5)	35 ± 14 (17–72)	38 ± 15 (19–77 )
Enhancement (%)				
* Geographical effect*	3 ± 0.7 (2–5)	5 ± 0.7 (3–6)	19 ± 8 (5–41)	15 ± 5 (5–29)
* Thermal acclimation*	−5 ± 0.4 (−6, −4)	3 ± 0.6 (2–4)	8 ± 6 (2–29)	6 ± 4 (2–20)

Individual geographical and thermal acclimation effects and enhancement were estimated with Eqns 6 and 7 respectively. Values correspond to global mean ± SD values across the 22 simulations and range (minimum–maximum) of values obtained across simulations.

**Figure 7:**
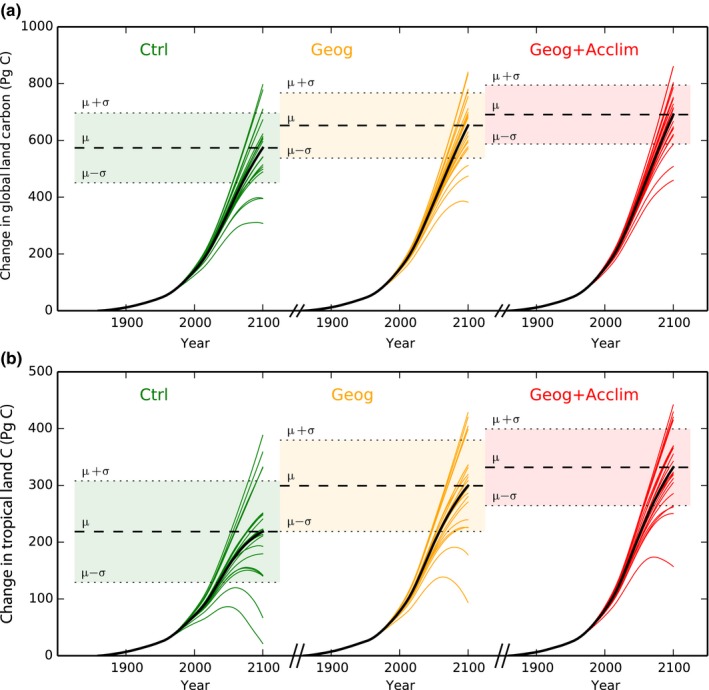
Simulated changes (respect to 1860) in (a) global terrestrial carbon stocks (Pg C) and (b) tropical land carbon under future climate change for *Ctrl*,* Geog* and *Geog+Acclim* simulations.

Finally, accounting for thermal acclimation of photosynthetic capacity narrows the range of predictions of the simulated global land C storage in 2100 across climate projections. Specifically, the variance of mean (σ^2^) land C accumulation in 2100 across models was reduced by 29% in *Geog+Acclim* and by 13% in *Geog* (Fig. 7a; Table S3 for values of σ^2^) compared to the *Ctrl*. This reduction is specifically due to a reduction in simulated uncertainty in the tropical region (Fig. 7b) with a 43% reduction in the variance of the regional mean across models with thermal acclimation and 19% due to geographical effects.

## Discussion

Our results for all model configurations suggested an overall increase in land carbon (C) sequestration in all three regions under future climate and CO_2_ conditions in line with earlier studies (e.g. Sitch *et al*., 2008). Importantly, the inclusion of geographical variation in the temperature response of photosynthetic capacity due to longer term processes (*Geog*) improved the model's ability to reproduce present‐day ecosystem‐level eddy‐covariance fluxes of gross primary productivity (GPP). Separating the geographical (*Geog* – *Ctrl*) and acclimation effects (*Geog+Acclim* minus *Geog*) in the KK07 formulation, allowed quantification of the individual effects (Table 1). Results demonstrated that there was a larger enhancement in the land C sink due to geographical effects than due to acclimation effects during the study period (Table 3). Inclusion of thermal acclimation became most relevant under future climate leading to an additional enhancement in the land sink in the tropics and temperate regions, which further increased ecosystem resilience to climate change (Table 3). However, although the future sink in boreal and tundra regions also benefitted from CO_2_ fertilization, this region did not benefit from thermal acclimation of photosynthetic capacity – at least, not as it was represented in this model. This is in agreement with results obtained in whole tree chamber experiments under elevated CO_2_ and temperature on Norway Spruce, a dominant Boreal forest species in which there was no stimulation of photosynthesis and growth with increased warming under natural low fertility conditions (Sigurdsson *et al*., 2013; Wallin *et al*., 2013). However, CO_2_ stimulation was obtained when soil fertility was improved. This is in contrast to results obtained on an old field‐grown Scots Pine on a nonfertile sandy soil on which elevated CO_2_ and temperature stimulated growth and photosynthesis (Wang *et al*., 1995; Peltola *et al*., 2002). In addition, Kroner & Way (2016) also obtained a reduction of leaf‐level net photosynthetic uptake under elevated CO_2_ and extreme temperatures in Norway Spruce seedlings grown in a glasshouse under well‐watered and ‐fertilized soils and high air humidity conditions. The authors identified photosynthetic capacity and not stomatal limitation or respiratory costs as the main mechanism behind the inability in Norway Spruce to maintain high levels of carbon gain at the high temperatures predicted for this region. Reduced photosynthetic capacity due to warming also is known to reduce growth and photosynthesis in Black spruce seedlings when grown under high soil fertility and well‐watered and high air humidity conditions (Way & Sage, 2008) for which there is little evidence for CO_2_ stimulation (Girardin *et al*., 2016). Taken together these results demonstrate the need for a better understanding of impacts of elevated temperature and CO_2_ on individual components of the temperature response of photosynthesis and growth for dominant boreal forest species.

Our results are in agreement with the modelling study by Chen & Zhuang (2013) on forest ecosystems in the US. Using an adaptation of the KK07 algorithms in the terrestrial ecosystem model (TEM), which does not incorporate modifications of the *J*_max_ to *V*_cmax_ (J : V) ratio with *T*_growth_, these authors obtained increased C uptake in the temperate region but a decrease in the boreal region under future climate conditions. However, our results contrast with those of Lombardozzi *et al*. (2015) and Smith *et al*. (2016) who both found a negative impact of thermal acclimation of photosynthetic capacity in the tropics and a positive impact on the arctic regions under future climate change conditions. The difference across studies is driven by the way acclimation of the J : V ratio is implemented; these authors implemented the acclimation effect on the J : V ratio by reducing *J*_max_ at 25°C. As a result, photosynthesis declines with increasing *T*_growth_, having negative effects of thermal acclimation on C storage in the tropics. In the present study, we implemented the decreasing J : V ratio by decreasing *J*_max_ and increasing *V*_cmax_ at the same time (as obtained by Lin *et al*., 2013), under the constraint that the total amount of leaf nitrogen is held constant. In our implementation, photosynthesis did not automatically decline with increasing *T*_growth_, resulting in a positive impact of thermal acclimation in tropical climate. However, at low *T*_growth_, our implementation also resulted in lower *J*_max_ at 25°C than in their studies, and we therefore obtained a negative effect of thermal acclimation in the boreal and tundra regions. Together these three studies provide a range in responses across dynamic global vegetation models (DGVMs) and across climate scenarios of the possible effects of thermal acclimation of photosynthetic capacity. This large range in responses highlights the urgent need for more analysis and data on the individual relationships of *V*_cmax_ and *J*_max_ at 25°C with *T*_growth_. All of these modelling results taken together should be considered as sensitivities that highlight the urgent need to understand what drives the J : V relationship to *T*_growth_ and how it should be implemented into models, that is, by varying *V*_cmax_ at 25°C, *J*_max_ at 25°C or both and by how much.

Significant uncertainties remain, however. Although the work by KK07 still represents the current state‐of‐the‐art in attempts to quantify the effects of thermal acclimation and geographical variation in the temperature sensitivity of photosynthesis, it nonetheless suffers from a number of shortcomings. One issue is that the relationships were largely derived for plants growing under CO_2_ concentrations in the range 350–400 ppm. We have assumed that the acclimation potential of plants does not change with rising CO_2_.

However, under elevated CO_2_ and temperature conditions, stomatal conductance is likely to decline therefore affecting leaf temperature with possible reductions on photosynthesis which might affect acclimation of *J*_max_ and *V*_cmax_ at elevated CO_2._ However, there is little information on leaf temperatures and how they are likely to change under future warming and CO_2_ conditions. There is an urgent need to monitor *T*_Leaf_ under current climate conditions but also in experiments under elevated CO_2_ and temperature, in parallel with physiological measurements. The literature on thermal acclimation of photosynthetic capacity mostly provides information on leaf‐level photosynthesis and/or plant growth at ambient CO_2_ concentrations; there is limited information reported under both high temperature and high CO_2_ conditions (Smith & Dukes, 2013). However, this assumption is supported by Crous *et al*. (2013), who found similar acclimation patterns of *J*_max_ and *V*_cmax_ at both ambient and elevated CO_2_ concentrations.

A second important issue is that the KK07 relationships do not differentiate between measurements under natural and experimental conditions, thus do not separate geographical and acclimation effects. By assuming the same algorithm, but applied in a different way, for the two effects, it allows a first attempt to quantify their individual contributions to the C cycle. For leaf dark respiration, Vanderwel *et al*. (2015) found that thermal acclimation can explain the observed geographical variation at ambient growth temperatures across the globe. Our study urgently calls for studies on temperature responses on photosynthetic capacity to focus separately on geographical variation due to long‐term processes and short‐term thermal acclimation, but also to carefully establish methodologies to separate them. A well‐known method to separate the geographical component is to carry out experiments on which species of different geographical origin are grown in an array of common temperature environments that span the thermal range of the species (Drake *et al*., 2017, and references therein). Specifically, our results call for the need for a comprehensive dataset on the geographical variability in the temperature sensitivity of photosynthetic capacity (i.e. *T*_opt,V_ and *T*_opt,J_ but also J : V ratio) to create equations suitable for DGVM implementation.

Third, the dataset assembled by KK07 is heavily weighted towards temperate species, with only two boreal species and no tropical species included. We have assumed that the relationships are valid for all three biomes. For boreal tree species, there is considerable additional evidence for thermal acclimation of photosynthesis (e.g. Way & Sage, 2008). Sendall *et al*. (2015) demonstrate similar acclimation capacity between boreal and temperate forest species in a warming experiment in Minnesota. For tropical tree species, however, the capacity for thermal acclimation of photosynthesis is very poorly quantified. There is clear evidence for geographical variation, with higher optimum temperatures for net photosynthetic uptake, *J*_max_ and *V*_cmax_ in warm‐adapted rainforest species than cool‐adapted species (Cunningham & Read, 2003; Vårhammar *et al*., 2015). Cunningham & Read (2003) observed acclimation of photosynthesis in a range of rainforest species exposed to temperatures up to 30°C, but found smaller acclimation capacity in tropical than temperate species and suggested that this was because tropical species are typically exposed to a smaller annual range of temperatures. The very limited data from warming experiments with tropical species to date show reductions in photosynthetic rate with long‐term warming (Doughty, 2011; Cheesman & Winter, 2013; Scafaro *et al*., 2017), possibly indicating a lack of acclimation capacity. However, these experiments used only individual leaves or seedlings in growth chambers; there is a very great need for realistic warming experiments in the field (Zhou *et al*., 2013; Cavaleri *et al*., 2015). The acclimation experiment on tropical seedlings by Slot & Winter (2017) found that these plants can acclimatize to moderate warming; however, photosynthesis decreases under high levels of warming. The study also reports that under elevated CO_2_ and warming sapling growth is stimulated.

Finally, we also note that the dataset of KK07 includes C_3_ species only. It has been estimated that nearly one‐quarter of total global plant photosynthesis is via the C_4_ pathway (Still *et al*., 2003), so there is a real need to incorporate temperature acclimation in C_4_ plants as well. However, there have been few studies of temperature acclimation in C_4_ plants expressed in terms of model parameters.

Our evaluation for present‐day conditions using eddy‐covariance derived GPP demonstrates that inclusion of geographical effects improves model comparison against observations, whereas inclusion of acclimation effects made little difference. Despite shortcomings from the KK07 formulation mentioned above, we consider that this formulation is state‐of‐the‐art, as it was demonstrated here to improve temperature responses for the present day. Our study also has demonstrated the importance of geographical variation and thermal acclimation on future C‐cycle projection. However, the uncertainty in results from this and earlier studies highlight the urgent need to measure thermal acclimation responses in a variety of ecosystems, especially in the tropics.

In order to enable new information on temperature sensitivity of photosynthesis to be captured in models, we would like to stress four key points. First, it is important that studies on temperature sensitivity of photosynthesis address the underlying processes and parameters that contribute to the temperature response of photosynthesis, such as the relationships of *V*_cmax_ and *J*_max_ at 25°C and the J : V ratio with *T*_growth_, and the variation of *H*_a_, *H*_d_, Δ*S* and *T*_opt,V_ and *T*_opt,J_ with *T*_growth_ on short timescales (days, months, seasons) and geographically. Without this information on underlying processes, it is difficult to extract information needed for current process‐based models. Second, we would like to emphasize the need to publish underlying datasets in order to facilitate comparison of parameters across experiments. Values of model parameters such as *V*_cmax_ and *J*_max_ depend strongly on the assumptions used when extracting them from data (Medlyn *et al*., 2002; Rogers *et al*., 2017) meaning that syntheses across experiments need access to the underlying data to ensure that values are comparable. Third, there also is a need to understand variations in stomatal conductance and foliar respiration with *T*_growth_, both geographically and temporally. Thus, measurements to determine the biochemical parameters listed above should be conducted in parallel with measurements of leaf respiration, and also stomatal conductance and photosynthesis taken under different vapour pressure deficit conditions under various *T*_growth_. Fourth, there is also an urgent need to go beyond measuring thermal acclimation of leaf photosynthesis and respiration only but also measure thermal acclimation of plant growth in order to assemble thermal responses of photosynthetic and respiration fluxes together with correspondent growth responses.

In conclusion, in this study, we have brought novel insights to the individual contribution of thermal acclimation and geographical variation of photosynthetic temperature responses to terrestrial C stores over different regions and temporal scales. Accounting for geographical variation was found to be most important under pre‐industrial and present‐day conditions while accounting for thermal acclimation becomes most relevant at elevated future temperatures. Acclimation also reduces the sensitivity of climate–carbon cycle models to climate change and therefore reduces the spread in global climate model projections. In the tropics, some existing models suggest that warming could eventually reduce land C storage. Finally, this study highlights the urgent need for more studies to aid refinement of modelled geographical variation and acclimation of thermal sensitivity of photosynthesis, especially in tropical regions where there is a paucity of data and large‐scale experiments with warming and elevated CO_2_ treatments. We also suggest that the modelling of plant responses to warming should become a much higher priority in global vegetation and Earth system modelling.

## Author contributions

L.M.M. and B.E.M. designed the research with contributions from C.H., S.S. and P.M.C.; L.M.M. and C.H. performed the simulations and L.M.M. performed the analysis; R.O.J. and C.H. performed model evaluation with valuable contributions from A.H. and D.C.; J.K. advised on implementation of acclimation algorithms; C.H. and P.Z. contributed to development of the IMOGEN tool; and L.M.M. wrote the manuscript with valuable contributions from all coauthors.

## Supporting information

Please note: Wiley Blackwell are not responsible for the content or functionality of any Supporting Information supplied by the authors. Any queries (other than missing material) should be directed to the *New Phytologist* Central Office.

**Fig. S1 **Optimum temperatures (*T*_opt_) for *V*_cmax_ and *J*_max_ and *J*_max_ to *V*_cmax_ ratio at 25°C.
**Fig. S2** Simulated Rubisco and electron transport limited light‐saturated sunlit leaf photosynthesis under *Geog+Acclim* and *Geog* under 2100 conditions for a boreal gridbox.
**Fig. S3 **Regression coefficients (*β*) values of linear regression (Eqn 7) obtained between observed and simulated GPP under the three configurations *Ctrl*,* Geog* and *Geog+Acclim*.
**Table S1** Parameters (‘*a’* and ‘*b’*, in Eqn S1) derived by Kattge & Knorr (2007).
**Table S2** Information on Fluxnext 2015 (22 sites) and Brasilflux sites (four sites) used for ecosystem‐level model evaluation
**Table S3** Global mean and SD (μ ± σ) fields at the end of 1860 and 2100 (including variance σ^2^) and change in global land carbon
**Notes S1** Details of JULES model plant physiology.
**Notes S2** Details of JULES–IMOGEN framework.
**Notes S3** Calculation of leaf‐level photosynthetic temperature responses.
