# Supplementary material for: Large sensitivity in land carbon storage due to geographical and temporal variation in the thermal response of photosynthetic capacity
Source: New Phytol. 2018 Apr 10;218(4):1462–77. doi: 10.1111/nph.15100 (PMC5969232; doi:10.1111/nph.15100)
Supplement: Supplementary file 1 — Fig. S1 Optimum temperatures (T opt) for V cmax and J max and J max to V cmax ratio at 25°C. Fig. S2 Simulated Rubisco and electron transport limited light‐saturated sunlit leaf photosynthesis under Geog+Acclim and Geog under 2100 conditions for a boreal gridbox. Fig. S3 Regression coefficients (β) values of linear regression (Eqn 7) obtained between observed and simulated GPP under the three configurations Ctrl, Geog and Geog+Acclim. Table S1 Parameters (‘a’ and ‘b’, in Eqn S1) derived by Kattge & Knorr (2007). Table S2 Information on Fluxnext 2015 (22 sites) and Brasilflux sites (four sites) used for ecosystem‐level model evaluation Table S3 Global mean and SD (μ ± σ) fields at the end of 1860 and 2100 (including variance σ2) and change in global land carbon Notes S1 Details of JULES model plant physiology. Notes S2 Details of JULES–IMOGEN framework. Notes S3 Calculation of leaf‐level photosynthetic temperature responses. [file NPH-218-1462-s001.pdf]

***New Phytologist* Supporting Information**

Article title: Large sensitivity in land carbon storage due to geographical and temporal variation in the thermal response of photosynthetic capacity

Authors: Lina M. Mercado, Belinda E. Medlyn, Chris Huntingford, Rebecca Oliver, Douglas B. Clark, Stephen Sitch, Przemyslaw Zelazowski, Jens Kattge, Anna B. Harper & Peter M. Cox.

Article acceptance date: 08 February 2018

The following Supporting Information is available for this article:

Notes S1 Details of JULES model plant physiology

Notes S2 Details of JULES-IMOGEN framework

Notes S3 Calculation of leaf level photosynthetic temperature responses

Table S1 Parameters (‘*a*’and ‘*b*’, in Eqn S1) derived by Kattge & Knorr (2007).

Table S2 Information on Fluxnext 2015(22 sites) and Brazilflux sites* (4 sites) used for ecosystem level model evaluation.

Table S3 Global mean and standard deviation ($\mu \pm \sigma$) fields at the end of 1860 and 2100 (including variance σ^2) and change in global land carbon.

Fig. S1 Optimum temperatures (T_{opt}) for V_{cmax} and J_{max} and J_{max} to V_{cmax} ratio at 25°C.

Fig. S2 Simulated Rubisco and light limited leaf photosynthesis under *Geog+Acclim* and *Geog* under 2100 conditions for a boreal gridbox.

Fig. S3 Regression coefficients (β) values of linear regression (Eqn 7) obtained between observed and simulated GPP under the three configurations *Ctrl*, *Geog* and *Geog+Acclim*.

Notes S1 Details of JULES model plant physiology: Photosynthesis model equations, leaf to canopy to grid level scaling, dynamic vegetation, stomatal conductance, leaf and plant respiration in JULES

Following Kattge & Knorr (2007), we used the Farquhar *et al.,* (1980) leaf C_3 photosynthesis model as described in Medlyn *et al., (*2002). Accordingly, the net photosynthetic uptake (A_n) in [mol m^{-2} s^{-1}] was calculated as the minimum of two limiting rates: Rubisco limited (A_v) or electron transport limited (A_J) photosynthetic uptake both in [mol m^{-2} s^{-1}] following Eqn S1 with R_d being the rate of leaf respiration.

$$A_n = min\{A_v, A_J\} - R_d \qquad \text{Eqn S1}$$

Rubisco limited photosynthesis is described by Eqn S2 where V_{cmax} in [mol m^{-2} s^{-1}] is the maximum rate of carboxylation of Rubisco, C_i and O_i are the intercellular concentrations of CO_2 and O_2 in [Pa], K_c and K_o in [Pa] are Michaellis Menten coefficients for Rubisco carboxylation and oxygenation respectively and Γ in [Pa] is the CO_2 compensation point in the absence of mitochondrial respiration.

$$A_v = \frac{V_{cmax}(C_i - \mathrm{T})}{\left[C_i + K_c\left(1 + \frac{O_i}{K_o}\right)\right]}$$

Eqn S2

Light limited photosynthesis is represented by Eqn S3 where J in [mol m^{-2} s^{-1}] is the rate of electron transport represented by Eqn S4 with a dependency on incident photosynthetically active photon flux density Q in [mol quanta m^{-2} s^{-1}], the potential electron transport J_{max} in [mol m^{-2} s^{-1}], the quantum yield of electron transport, α in [mol electrons mol $^{-1}$ photon] and a curvature factor, θ [unitless].

$$A_J = \left(\frac{J}{4}\right)\frac{(C_i - \mathrm{T})}{(C_i + 2\mathrm{T})}$$

Eqn S3

$$\theta J^2 - (\alpha Q + J_{max})J + \alpha Q J_{max} = 0 \qquad \text{Eqn S4}$$

Eqn 1 in the main document represents the temperature dependency for J_{max} and V_{cmax}. The temperature dependency of K_c, K_o, and Γ were taken from Bernacchi *et al.* (2001) as described in Medlyn *et al.* (2002).

A soil moisture dependence is included in the net photosynthesis term, thus indirectly affecting the calculation of internal CO_2 concentration and stomatal conductance (Best *et al.,* 2011). Eqn S5 relates the ratio of leaf intercellular (C_i) to external CO_2 concentration (C_a) in [Pa] to leaf humidity deficit (D) in [Kg H2O/ Kg air], CO_2 compensation point (Γ) in [Pa] and two plant functional type (PFT) specific empirical constants (f_o and D_o) [unit-less], which are directly related to the Leuning *et al.,* (1995) stomatal conductance model. Stomatal conductance (g_s) in [m s^{-1}] and net leaf photosynthetic uptake (A_n) in [mol m^{-2} s^{-1}] are then linked via the CO_2 diffusion equation (S6).

$$\frac{C_i - \Gamma}{C_a - \Gamma} = f_0 \left(1 - \frac{D}{D_0}\right)$$

Eqn S5

$$A_n = g_s(C_a - C_i)/1.6$$ Eqn S6

Leaf respiration at any temperature is calculated as a function of V_{cmax} at the respective temperature following Eqn S7 where F_d [unitless] is a PFT dependent parameter.

$$R_d = F_d x V_{cmax}$$ Eqn S7

In this study, scaling from leaf to canopy level (photosynthesis, respiration and stomatal conductance) was done using the big-leaf approach option within the JULES model. Canopy level flux was estimated the integral of the leaf level individual processes over the entire canopy leaf area. Remaining plant respiration components were estimated as a function of canopy respiration and individual tissue N:C ratios. Therefore, all plant respiration components retain the temperature response function of V_{cmax}. PFT specific parameters for biochemistry,

photosynthesis and stomatal conductance are given in Table 2 of Clark *et al.* (2011). Scaling to ecosystem level or grid box level was done by adding the individual contributions (fluxes and stocks) of each PFT weighted by their gridcell fractional coverage. Competition between PFTs (broadleaf trees, needle-leaf trees, C_3 and C_4 grasses, and shrubs), i.e. dynamic vegetation, is included in all simulations. Competition is based on a prescribed dominance hierarchy -tree-shrub-grass – where dominant PFTs limit expansion of subdominant PFTs. Furthermore, JULES simulates a surface energy balance and includes a calculation of skin (leaf) temperature. Finally, calculations of photosynthesis, respiration, and full energy balance were done at hourly time scale and the vegetation dynamics module was updated every ten days.

Notes S2 Details of JULES-IMOGEN framework

Mean warming was calculated as a function of radiative forcing (via a parameterised energy balance model, EBM), which in turn is dependent on any altered atmospheric gas composition (Huntingford *et al.,* 2010). Therefore, changes in the terrestrial carbon storage feedback on climate via atmospheric carbon dioxide (CO_2) concentration. This is in addition to the changes in CO_2 concentration directly due to fossil fuel burning, or draw-down into the oceans. The oceanic uptake of atmospheric CO_2 was calculated for each year, based on atmospheric CO_2 concentration and temperature changes since pre-industrial, and up to that year, using an impulse response function. This was calibrated against the Princeton 3-D biogeochemical ocean model following Joos *et al.* (1996) as documented in Huntingford *et al.* (2004).

Notes S3 Calculation of leaf level photosynthetic temperature responses (Fig. 1)

We calculated the leaf-level temperature response of gross photosynthesis using the Farquhar *et al.* (1980) model (Eqns S1-S4) and the KK07 algorithms (Eqns 1-5) for 1860 (pre-industrial) and 2100 (future) climatic conditions for the three model configurations under mean cold season (e.g. mean over

winter/spring/autumn) and summer conditions for the two high latitude locations and mean of all months for the one tropical location. Input consisted of atmospheric CO_2 and T_{growth} over the study seasons for the two years, 1860 and 2100, at the three locations. Additionally, we assumed constant levels of light saturation of photosynthesis for each PFT (Table 2). For year 1860 we used pre-industrial global CO_2 concentration (Le Quere *et al.,* 2016) and for 2100 we used the predicted values from the global JULES-IMOGEN for each of the three configurations, using climate model patterns and energy balance model parameters from a GCM with intermediate levels of predicted warming (gfdl_cm2). T_{growth} was used to calculate $T_{opt,V}$ and $T_{opt,J}$ and JV following KK07 as outlined above. The initial V_{cmax} at 25°C was estimated as in standard JULES from top of the canopy Nitrogen levels (Clark *et al.,* 2011). T_{growth} for the *Geog* configuration was based on the monthly pre-industrial air temperatures extracted from the CRU dataset at the three locations, as explained in the methods. By definition of the *Geog* configuration, T_{growth} is the same in 1860 and 2100. T_{growth} in *Geog+Acclim* was calculated for years 1860 and 2100 as the mean monthly air temperature of the months covered in the respective seasons for the two high-latitude gridcells and as the mean over the whole year for the tropical gridcell. Monthly air temperatures at pre-industrial (1860) were taken from the CRU data set (New *et al.,* 2000) and for 2100 extracted from the global JULES-IMOGEN simulation with the *Geog+Acclim* configuration, for each of three locations. Also, for computational simplicity, and only for these leaf-level photosynthetic temperature responses, internal CO_2 concentration (C_i) was prescribed as 70% of the atmospheric CO_2. We note that the optimal temperatures were likely overestimated as a result, since increasing vapour pressure deficit (VPD) with temperature is likely to drive stomatal closure (Lin *et al.,* 2012); changing VPD was however accounted for in all the full global JULES-IMOGEN simulations. We used this information to produce temperature response curves over a range of relevant temperatures at each location.

To show the implication of the leaf-level results at the regional scale we extracted the Gross Primary Productivity (GPP) for all gridcells for three regions, tropical (30°N <Lat <30°S), temperate (60°N >Lat >30°N and 60°S >Lat > 30°S) and boreal

and tundra (60^oS < Lat >60^oN) regions from the global JULES simulations for PI and 2100 forced with the gfdl_cm2 climate, i.e. same model used in the leaf level temperature responses described above.

Table S1 Parameters (a and b, in Eqn S8) derived by Kattge & Knorr (2007) and used in this study to parameterize spatial and temporal variation of temperature responses of J_{max} , V_{cmax} and their ratio with T_{growth}.

	a	*b*
J_{max}	659.70	-0.75
V_{cmax}	668.39	-1.07
J_{max} / V_{cmax}	2.59	-0.035

$$x_i = a_i + b_i \times T_{growth} \qquad \text{Eqn S8}$$

Where ‘x’ can be ΔS or JV ratio, and ‘i’ denotes J_{max}, V_{cmax} and JV.

Table S2

Information on Fluxnext 2015(22 sites) and Brasilflux sites* (4 sites) used for ecosystem level model evaluation. Vegetation Types correspond to Evergreen Broadleaved forest & Evergreen Needle leaved Forest (ENF & NT), Deciduous Broadleaved forest (DBF), Mixed forest sites and Mixed forest sites with 70% broad leaved tree coverage (MF,) and C_3 grasses (C3G). The fractional coverage of soil and each vegetation type as represented by Jules five plant functional types is also given: broad leaved (BT), needle leaved (NT), C_3 and C_4 grasses and Shrubs (SH).

Site	Timestep (s)	Startyear	Endyear	Vegetation Type	Latitude	Longitude	BT	NT	C_3	C_4	SH	soil
AT_Neu	1800	2002	2012	C3G	47.12	11.32	-	-	0.8	-	-	0.2
CA_Oas	1800	1996	2010	MF	53.5	-106.20	0.35	0.35	0.2		-	0.1
CG_Tch	1800	2006	2009	MF	-4.5	11.66	0.5	-	-	0.15	0.25	0.1
CH_Cha	1800	2006	2014	C3G	47.21	8.41	-	-	0.8	-	-	0.2
CN_HaM	1800	2002	2004	C3G	37.37	101.18	-	-	0.8	-	-	0.2
DE_Tha	1800	1996	2014	NT	51	13.57	-	1	-	-	-	-
FI_Hyy	1800	1996	2014	NT	62	24.30	-	1	-	-	-	-
GF_Guy	1800	2004	2014	EBF	5.28	-52.92	1	-	-	-	-	-
IT_CA1	1800	2011	2014	DBF	42.38	12.02	1	-	-	-	-	-
IT_Col	1800	1996	2014	DBF	42	13.59	1	-	-	-	-	-
IT_Ren	1800	1998	2013	NT	46.6	11.43	-	1	-	-	-	-
RU_Che	1800	2002	2005	C3G	68.5	161.34	-	-	0.8	-	-	0.2
RU_SkP	1800	2012	2014	DNF	62.26	129.17	0.1	0.9	-	-	-	-
US_Ha1	3600	1991	2012	DBF	42.5	-72.17	1	-	-	-	-	-
US_MMS	3600	1999	2014	DBF	39.5	-86.41	1	-	-	-	-	-
US_Pfa	3600	1995	2014	MF	45.95	-90.27	0.7	0.3	-	-	-	-
US_SRM	1800	2004	2014	MF	31.8	-110.87	-	-	-	0.55	0.35	0.1
US_Ton	1800	2001	2014	MF	38.5	-120.97	0.3	-	0.3	-	0.3	0.1
US_UMB	3600	2000	2014	DBF	45.56	-84.71	1	-	-	-	-	-
US_Var	1800	2000	2014	C3	38.41	-120.95	0.2	-	0.75	-	-	0.05
US_Whs	1800	2007	2014	MF	31.74	-110.05	-	-	-	0.1	0.8	0.1
US_WCr	1800	1999	2014	DBF	45.8	-90.08	1	-	-	-	-	-
LBA_BAN*	3600	2004	2007	EBF	-9.82	-50.16	1	-	-	-	-	-
LBA_K34*	3600	2003	2006	EBF	-2.61	-60.21	1	-	-	-	-	-
LBA_K83*	3600	2001	2004	EBF	-3.05	-54.97	1	-	-	-	-	-
LBA_RJA*	3600	2000	2003	EBF	-10.08	-61.93	1	-	-	-	-	-

Table S3

Global mean and standard deviation ($\mu \pm \sigma$) fields at the end of 1860 and 2100 (including variance σ^2) and change in global land carbon estimated as values in 2100 minus 1860. All model simulations - using patterns of climate from 22 global climate models and 3 model configurations- were forced with the same climatology during spin up period. Global Land Carbon is the total amount of carbon in soil and vegetation, enhancement was estimated with Eqn (9).

1860	$\mu \pm \sigma$	2100	$\mu \pm \sigma$	σ^2
**Global Land C [Pg]**		**Global Land C [Pg]**		
Control	1422± 0.01	*Control*	1995.7± 123	15102.2
Geog	1514± 0.01	*Geog*	2166.0 ± 115	13184.5
Geog+ Acclim	1546 ± 0.01	*Geog+ Acclim*	2236.4 ± 104	10747.1
**Enhancement [%]**		**Tropical Land C [Pg]**		
Geographical effects	6.4%	*Control*	839.7 ± 89	7979
Thermal acclimation	2.1%	*Geog*	993.5 ± 80	6468
		Geog+ Acclim	1026.8 ± 67	4561
**Global GPP [Pg]**				
Control	119 ± 0.007			
Geog	119 ± 0.006			
Geog+ Acclim	120 ± 0.007			

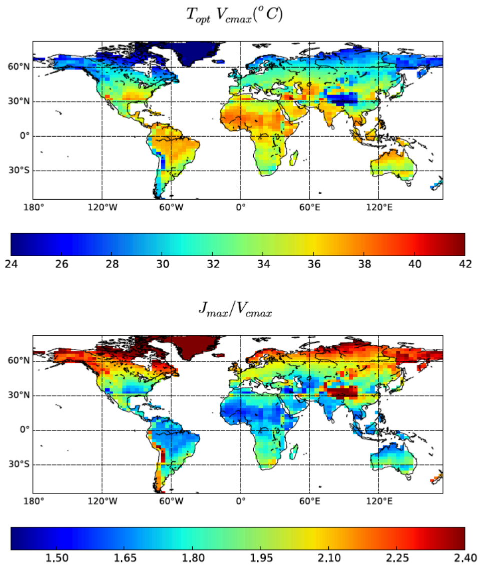

Figure S1

Optimum temperatures (T_{opt}) for V_{cmax} and J_{max} and J_{max} to V_{cmax} ratio at 25°C with T_{growth} estimated from the CRU data set (New et *al.,* 2000) for the growth periods of the years 1901-1910.

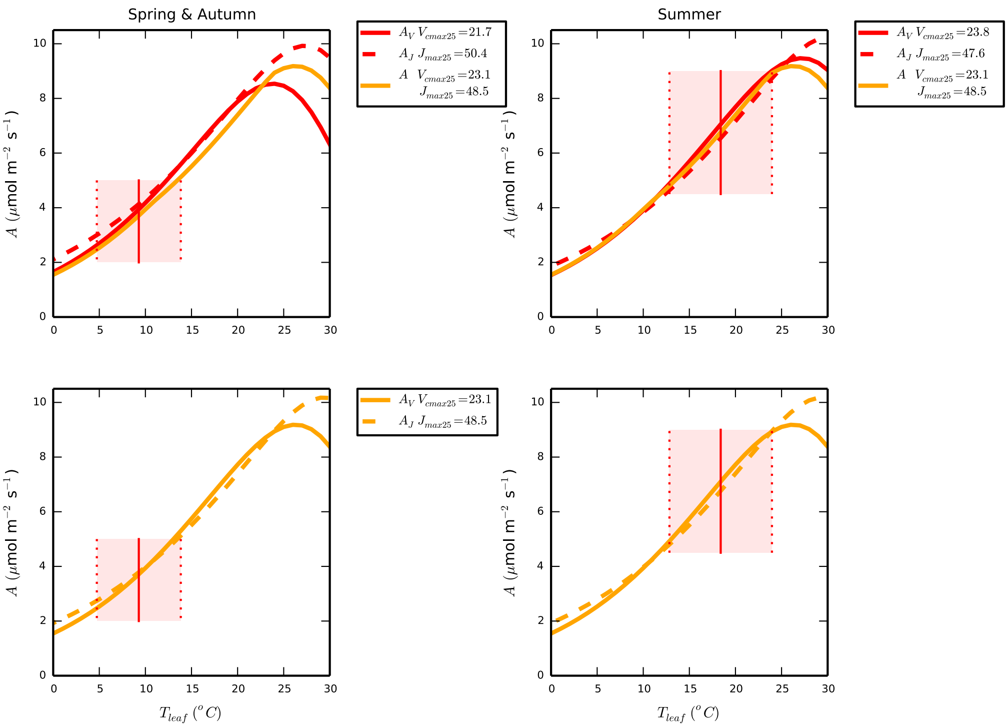

Figure S2

Simulated Rubisco (A_v) and light limited (A_j) light saturated sunlit leaf photosynthesis under *Geog+ Acclim* (top panel, red lines,) and *Geog* (lower panel, orange lines) under 2100 conditions for a boreal gridbox using growth and day-time leaf temperatures from the gfdl-cm2 model during autumn and spring (left panels) and summer months (right panels). During spring and autumn, there is Rubisco limitation to photosynthesis over the simulated day-time leaf temperature range (mean ± one standard deviation, $\mu \pm \sigma$ represented in the shaded red box). During summer, there is light limitation to photosynthesis over the simulated day-time leaf temperature range. Under *Geog+Acclim*, photosynthetic capacity at 25°C changes with increasing growth temperatures with V_{cmax}, increasing from 21.7 to 23.8 μmol m^{-2} s^{-1} and J_{max} declining from 50.4 to 47.6 μmol m^{-2} s^{-1}.

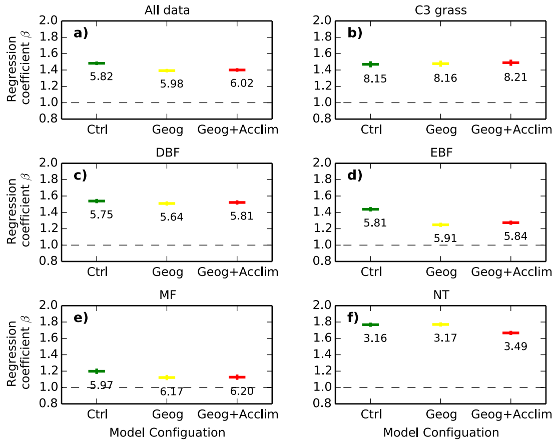

Figure S3

Regression coefficients (β) values of linear regression (Eqn 7) obtained between observed and simulated GPP under the three configurations *Ctrl*, *Geog* and *Geog+Acclim*. Annotations are the Root Mean Square Error (RMSE) and vertical lines correspond to 95 % confidence intervals. Vegetation Types correspond to C_3 Grass (C3G), Evergreen Broadleaved forest & Evergreen Needle leaved Forest (ENF & NT), Deciduous Broadleaved forest (DBF), Mixed forest sites (MF) with 70% broad leaved tree coverage and 30% C_3 grass.

References

Bernacchi CJ, Singsaas EL, Pimentel C, Portis Jr AR, Long SP. 2001.Improved temperature response functions for models of Rubisco-limited photosynthesis. *Plant, Cell & Environment* **24**:253–259.

Best MJ, Pryor M, Clark DB, Rooney GG, Essery R, Ménard CB, Edwards JM, Hendry MA, Porson A, Gedney N *et al.* 2011. The Joint UK Land Environment Simulator (JULES), model description–Part 1: energy and water fluxes. *Geoscientific Model Development* **4**:677–699.

Clark DB, Mercado LM, Sitch S, Jones CD, Gedney N, Best MJ, Pryor M, Rooney GG, Essery RL, Blyth E *et al.* 2011. The Joint UK Land Environment Simulator (JULES), model description–Part 2: carbon fluxes and vegetation dynamics. *Geoscientific Model Development* **4**:701–22.

Farquhar GD, von Cammerer S, Berry JA. 1980. A biochemical model of photosynthetic CO2 in leaves of C_3 species. *Planta* **149**:78–90.

Huntingford C, Harris PP, Gedney N, Cox PM, Betts RA, Marengo JA, Gash JH. 2004. Using a GCM analogue model to investigate the potential for Amazonian forest dieback. *Theoretical and Applied Climatology*. **78**:177–85.

Huntingford C, Booth BB, Sitch S, Gedney N, Lowe JA, Liddicoat SK, Mercado LM, Best MJ, Weedon GP, Fisher RA *et al.* 2010. IMOGEN: an intermediate complexity model to evaluate terrestrial impacts of a changing climate. *Geoscientific Model Development* **3**:679–687.

Joos F, Bruno M, Fink R, Siegenthaler U, Stocker TF, Le Quere C, Sarmiento JL. 1996. An efficient and accurate representation of complex oceanic and biospheric models of anthropogenic carbon uptake. *Tellus B: Chemical and Physical Meteorology* **48**:394–417.

Kattge J, Knorr W. 2007. Temperature acclimation in a biochemical model of photosynthesis: a reanalysis of data from 36 species. *Plant, Cell & Environment* **30**: 1176–1190.

Le Quéré C, Andrew RM, Canadell JG, Sitch S, Korsbakken JI, Peters GP, Manning AC, Boden TA, Tans PP, Houghton RA *et al.* 2016. Global carbon budget 2016. *Earth System Science Data* **8**:605–649.

Leuning R. 1995. A critical appraisal of a combined stomatal-photosynthesis model for C_3 plants. *Plant, Cell & Environment* **18**:339–355.

Lin YS, Medlyn BE, Ellsworth DS. 2012. Temperature responses of leaf net photosynthesis: the role of component processes. *Tree Physiology* **32**:219–231.

Medlyn BE, Dreyer E, Ellsworth D, Forstreuter M, Harley PC, Kirschbaum MU, Le Roux X, Montpied P, Strassemeyer J, Walcroft A, Wang K. 2002.

Temperature response of parameters of a biochemically based model of photosynthesis. II. A review of experimental data. *Plant, Cell & Environment* **25**:1167–79.

New M, Hulme M, Jones P. 2000. Representing twentieth-century space-time *clim*ate variability. Part II: Development of 1901–96 monthly grids of terrestrial surface climate. *Journal of Climate* **13**:2217–2238.
